# Binnensporten tijdens de COVID-19-pandemie: analyse met behulp van data uit de COVID RADAR app

**DOI:** 10.1007/s12508-022-00351-0

**Published:** 2022-06-07

**Authors:** Willian J. van Dijk, Nicholas H. Saadah, Mattijs E. Numans, Jessica C. Kiefte-de Jong

**Affiliations:** Afdeling Public Health en Eerstelijns Geneeskunde, Leids Universitair Medisch Centrum, Leiden, Nederland

**Keywords:** COVID-19, beleid, lockdown, sporten, COVID-19, Policy, Quarantine, Sports

## Abstract

Binnensportaccommodaties werden langer gesloten gehouden dan buitensportaccommodaties gedurende de lockdowns tijdens de COVID-19-pandemie. Dit onderzoek beantwoordt de vraag of binnen sporten het risico op besmetting met SARS-CoV‑2 vergroot. Hiervoor werden gegevens gebruikt van de COVID RADAR-app. Er werd gecorrigeerd voor leeftijd, vaccinatiestatus, geslacht, ander risicogedrag, prevalentie van SARS-CoV‑2 en kwaliteit van de leefomgeving. Uit analyses van 1.353 gebruikers, van wie 13,0% een positieve testuitslag rapporteerde, blijkt dat binnensporters significant vaker een positieve test hadden (oddsratio 1,8; 95%-betrouwbaarheidsinterval 1,2–2,6; *p* = 0,003) ten opzichte van gebruikers die buiten sporten. Dit bevestigt de rationale achter het eerdere beleid ten aanzien van het langer gesloten houden van binnensportaccommodaties om het aantal besmettingen te reduceren.

**Digitaal aanvullende content:**

De online versie van dit artikel (10.1007/s12508-022-00351-0) bevat aanvullend materiaal, toegankelijk voor daartoe geautoriseerde gebruikers.

## Abstract (en)

Indoor sporting locations have been forced to stay closed longer than outdoor sporting locations during the lockdowns introduced during the COVID-19 pandemic. This research addresses the question whether exercising indoors does raise one’s risk of infection with SARS-CoV‑2. This analysis was performed using data from the COVID RADAR app and accounted for user differences in age, vaccine status, sex, other risky behavior, SARS-CoV‑2 prevalence and quality of living environment. Analyzing data from 1,353 users, 13.1% of which reported a positive SARS-CoV‑2 test, we found that exercising indoors leads to an odds ratio of 1.8 with a positive SARS-CoV‑2 test (95%-confidence interval 1.2–2.6, *p* = 0.003), as compared to users who exercise outdoors. This confirms the justification used in longer closing indoor sporting locations to reduce the number of infections.

## Inleiding

Na de lockdowns gedurende de COVID-19 (COronaVIrus Disease 2019)-pandemie in Nederland werden buitensportaccommodaties eerder geopend dan binnensportaccommodaties. Het idee hierachter is ook nu nog dat de kans op een besmetting met SARS-CoV‑2 (severe acute respiratory syndrome coronavirus 2) binnen in een zaal of een sportschool groter is dan in buitensportaccommodaties. Bewijs daarvoor ontbreekt tot nu toe [1].

De COVID RADAR-app is een gratis smartphone-app waarin vragen gesteld worden over symptomen en gedrag (waaronder het houden van afstand en sportgedrag). Ook kunnen gebruikers aangeven dat ze onlangs getest zijn op SARS-CoV‑2 en wat de uitslag van die test was. In een eerdere publicatie hebben we beschreven dat de COVID RADAR-app een valide meetinstrument is voor surveillance van SARS-CoV‑2, waarbij incidentie van symptomen toeneemt rond een positieve test, en dat verplaatsings- en contactgedrag in de weken voor een positieve test bovengemiddeld hoog waren [2]. Gecombineerd met vragen over sporten van de gebruikers geeft de app een unieke mogelijkheid om een antwoord te krijgen op de vraag of de rationale achter het eerder overheidsbeleid met betrekking tot binnen sporten en COVID-19 klopt.

Dit verslag beantwoordt met behulp van data van de COVID RADAR-app de vraag of mensen die binnen sporten daadwerkelijk vaker besmetting met SARS-CoV‑2 rapporteren dan mensen die buiten sporten.

## Methode

Voor deze analyse werden data gebruikt van de COVID RADAR-app [2]. Meer dan 280.000 personen vulden de app in totaal meer dan 8,5 miljoen keer in over een periode van bijna 2 jaar (april 2020 tot en met februari 2022). Gebruikers worden aangemoedigd de app dagelijks in te vullen, maar zijn dat niet verplicht. Dit niet-verplichtende beleid resulteerde in een groep van 43.500 ‘trouwe’ gebruikers die meer dan 75% van de observaties leverden.

Omdat inzichten gedurende de tijd veranderden is in de bijna twee jaar dat de app in de lucht was, de inhoud enkele malen aangepast. Zo is na een half jaar de vraag over sporten toegevoegd (augustus 2020), kwam er in januari 2021 de mogelijkheid bij om een negatieve test te rapporteren en konden gebruikers aangeven of ze gevaccineerd waren. Gezien deze aanpassingen (maar ook vanwege veranderingen in het testbeleid) zijn in de huidige analyses alleen gerapporteerde testuitslagen van na 1 januari 2021 geïncludeerd.

Het theoretische beloop tussen besmetting en rapportage van een testuitslag in de app is op te delen in verschillende fasen. Ten eerste vertoont een individu risicogedrag, wat leidt tot virale transmissie. Vervolgens ontwikkelen zich na de incubatieperiode (gemiddeld 2–14 dagen) symptomen bij het geïnfecteerde individu. Deze maakt dan een afspraak voor een test op de aanwezigheid van SARS-CoV‑2 (1–3 dagen). Vervolgens wordt de test afgenomen en wordt de testuitslag bekend, die uiteindelijk in de app gerapporteerd wordt (2–3 dagen). Omdat het precieze moment van besmetting met SARS-CoV‑2 nooit met zekerheid vast te stellen is, gebruiken we een tijdsperiode van 10–20 dagen voor een testuitslag waarin het risicogedrag en dus ook de besmetting mogelijk heeft plaatsgevonden. Deze theoretische tijdsperiode wordt bevestigd in onze eerdere publicatie, waarin gemiddeld gedrag van gebruikers die uiteindelijk positief testen, hoger was in de tijdsperiode van 10–20 dagen voor een positieve test [2–4].

In de app konden gebruikers aangeven of ze onlangs getest waren en wat de uitslag van de test was. Voor deze analyse includeren we per gebruiker alleen eerste vermeldingen van een positieve of negatieve test. Personen die zowel een positieve als negatieve testuitslag rapporteerden konden tweemaal worden meegenomen in de analyse, onder voorwaarde dat deze tests minimaal 60 dagen na elkaar werden gerapporteerd.

In de app werd ook gevraagd of de gebruiker aan sport deed. De antwoordmogelijkheden waren: nee, ja (bij een vereniging of sportschool binnen), ja (bij een vereniging of sportschool buiten) of ja (ergens anders, zowel binnen als buiten). Voor het beantwoorden van de onderzoeksvraag selecteerden we alleen gebruikers die binnen de periode van mogelijke besmetting minimaal één keer hadden gesport, zowel binnen als buiten. Een gebruiker sportte binnen als er werd geantwoord: ‘ja, bij een vereniging of sportschool binnen’. Omdat personen die hadden geantwoord: ‘ja (ergens anders, zowel binnen als buiten)’ zowel binnen als buiten konden sporten, werden zij geëxcludeerd.

Omdat vaccinatiestatus de kans op het geïnfecteerd raken beïnvloedt, werd hiervoor gecorrigeerd. Een gebruiker is gevaccineerd als deze bij alle observaties in de mogelijke besmettingsperiode aangeeft volledig gevaccineerd te zijn. Leeftijdscategorieën werden gecategoriseerd in < 18 jaar, 19–49 jaar, 50–69 jaar en ≥ 70 jaar. Gender werd gecategoriseerd in man (1) en niet man (0), inclusief non-binaire genders.

Omdat sociale en regionale omgevingsfactoren de kans op besmetting met SARS-CoV‑2 beïnvloeden hebben wij hiervoor gecorrigeerd [5]. Daarvoor werd de Leefbarometer gebruikt – een score die per regio berekend wordt met behulp van meer dan 100 indicatoren uit zowel sociale als fysieke domeinen, die een maat geven van de kwaliteit van de leefomgeving [6].

Andere mogelijke factoren die de kans op besmetting met SARS-CoV‑2 beïnvloeden zijn andere vormen van risicogedrag en de periodeprevalentie van SARS-CoV‑2. Hiervoor hebben we gecorrigeerd door het gemiddelde van de antwoorden van de gebruiker op de vraag over het aantal personen binnen de 1,5 meter in het geselecteerde tijdvak van 10–20 dagen voor een positieve test te gebruiken. Om te corrigeren voor de SARS-CoV-2-prevalentie hebben we openbare data van het Rijksinstituut voor Volksgezondheid en Milieu (RIVM) gebruikt over het aantal dagelijks positieve SARS-CoV-2-tests per gemeente [7]. Om de periodeprevalentie te schatten werd het aantal positieve tests van drie dagen terug en vijf dagen in de toekomst opgeteld en als getal per 100.000 inwoners gerapporteerd. De periode van drie dagen voor een test en vijf dagen na een test is gekozen omdat de geteste persoon in die periode in theorie besmettelijk is. Vervolgens werd deze periodeprevalentie per gebruiker gemiddeld in het geselecteerde tijdvak. Beschrijvende analyses tussen gebruikers die wel en niet binnen sportten werden getoetst met de student T‑test voor continue variabelen en de chi-kwadraattoets voor categoriale variabelen.

Vervolgens werd er een *binomial generalized linear mixed model* gebruikt, met als uitkomstvariabele een positieve SARS-CoV-2-test, om verder het verband met sporten te onderzoeken. Om te corrigeren voor clustering als gevolg van herhaalde metingen, werd er een random *intercept* toegevoegd op individueel niveau. Effecten zijn gerapporteerd in de vorm van een oddsratio (OR) en robuuste 95%-betrouwbaarheidsintervallen (BI). Er werd gecorrigeerd voor leeftijd, vaccinatiestatus, gender, gemiddeld aantal personen binnen de 1,5 meter gerapporteerd, periodeprevalentie van SARS-CoV‑2 en mate van kwaliteit van de leefomgeving van de gebruiker.

In totaal werden drie analyses uitgevoerd: een hoofdanalyse naar het verband tussen minimaal één keer binnen sporten en een positieve test, en twee subanalyses. Omdat in de hoofdanalyse binnensporters ook buiten konden sporten werd in de eerste subanalyse dezelfde analyse uitgevoerd als bij de hoofdanalyse, maar in een andere gebruikersgroep, namelijk gebruikers die of alleen maar binnen sporten of alleen maar buiten sporten. De tweede subanalyse werd in dezelfde gebruikersgroep als de hoofdanalyse uitgevoerd, maar nu werd het verband tussen iedere dag extra binnen sporten en een positieve test getest. Analyses werden uitgevoerd in STATA 16.1 (Syntax is bijgevoegd in de digitaal aanvullende content, bijlage 1).

## Resultaten

**Figure 1:**
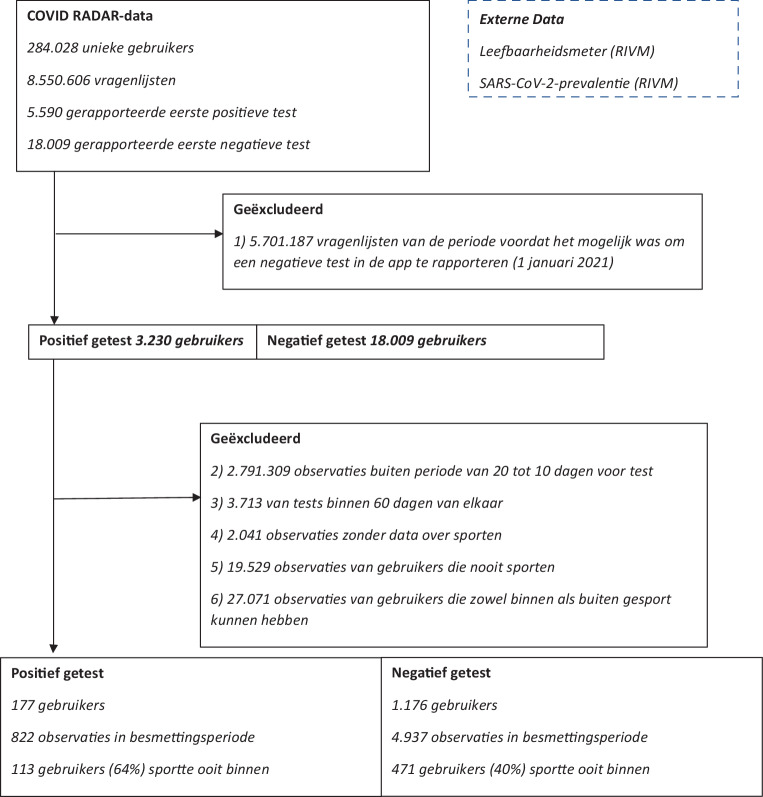

Ongeveer 50% van de COVID RADAR-app-gebruikers sportte minimaal eenmaal per week, wat overeenkomt met het gemiddelde in Nederland [8]. In totaal werden in de periode na 1 januari 2021 21.239 testuitslagen in de app gemeld (zie fig. 1). Van de gebruikers van wie de gegevens uiteindelijk geschikt waren voor de analyse (*n* = 1.353) sportte 43% ooit binnen. Dit is ook vergelijkbaar met Nederlandse statistieken [8]. Van hen testten 177 (13,1%) positief (zie digitaal aanvullende content, bijlage 2). COVID RADAR-app-gebruikers waren ouder dan gemiddeld in Nederland (zie tab. 1). Binnensporters waren vaker gevaccineerd (37% ten opzichte van 63%, *p* < 0,001) en hadden vaker de app ingevuld (gemiddeld 3,9 maal ten opzichte van 4,7 maal, *p* < 0,001). De kwaliteit van de leefomgeving was significant lager bij binnensporters (gemiddelde Z‑waarde 0,09 ten opzichte van 0,06; *p*-waarde 0,01). Het gemiddelde aantal personen binnen de 1,5 meter was hoger bij binnensporters (3,2 ten opzichte van 4,3; *p* < 0,001). De gemiddelde prevalentie van SARS-CoV‑2 ten tijde van het sporten was bij binnensporters hoger (376 inwoners per 100.000 ten opzichte van 644 per 100.000 inwoners, *p*-waarde < 0,001).

**Table 1:** 

Binnen sporten	Nee	%	Ja	%	*p*-waarde
0–18 jaar	48	64	27	36	
19–49 jaar	130	63,7	74	36,2	
50–69 jaar	457	55,6	365	44,4	
70+ jaar	134	53,1	118	46,8	
					0,06
Man	346	59,8	233	40,2	0,06
Gevaccineerd	228	37	388	63	< 0,001
Totaal	769	56,8	584	43,2	
	*gemiddelde*	*standaarddeviatie*	*gemiddelde*	*standaarddeviatie*	
Leefbarometer (Z-waarde)^a^	0,09	0,17	0,06	0,16	0,01
Aantal observaties per gebruiker	3,9	3,0	4,7	3,3	< 0,001
Gemiddeld aantal personen binnen de 1,5 meter	3,2	4,6	4,3	5,8	< 0,001
Gemiddelde prevalentie (per 100.000 inwoners)	376	575	644	990	< 0,001

^**a**^Z‑waarde ten opzichte van het Nederlands gemiddelde

**Figure 2:**
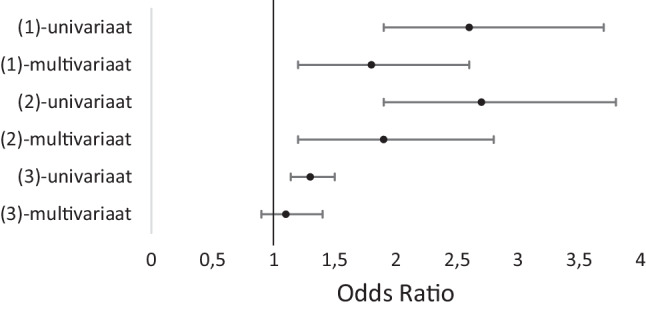

Van de gebruikers die alleen buiten hadden gesport was 8,3% van de testuitslagen positief. Van de gebruikers die minimaal één keer binnen hadden gesport was 19,4% van de testuitslagen positief (zie tab. 2). Na correctie voor leeftijd, geslacht, ander risicogedrag, SARS-CoV-2-periodeprevalentie, vaccinatiestatus en Leefbarometer was er een verband tussen minimaal één keer binnen sporten en een positieve testuitslag ten opzichte van gebruikers die alleen maar buiten sporten (zie fig. 2, OR: 1,78 (95%-betrouwbaarheidsinterval (95%-BI) 1,21–2,61; *p* = 0,003)).

**Table 2:** 

	Buiten sporten	Binnen sporten
Negatief	705 (91,7%)	471 (80,6%)
Positief	64 (8,3%)	113 (19,4%)
Totaal	769	584

In de eerste subanalyse met gebruikers die of alleen maar binnen sportten of alleen maar buiten sportten bleek in totaal 8,3% positief te testen na alleen buiten gesport te hebben ten opzichte van 19,7% na alleen binnen sporten (zie digitaal aanvullende content, bijlage 3). Na correctie bleek een OR van 1,84 (95%-BI 1,23–2,75; *p* = 0,003) (zie fig. 2).

In de tweede subanalyse waarbij het verband tussen aantal dagen binnen sporten en het hebben van een positieve test werd onderzocht, bleek na correctie voor de eerdergenoemde factoren dat iedere dag extra binnen sporten een OR had van 1,10 (95%-BI 0,90–1,35; *p* = 0,363) (zie fig. 2). (Zie digitaal aanvullende content, bijlage 4 en 5).

## Beschouwing

We vonden in de met de COVID RADAR-app verzamelde gegevens dat het binnen sporten samenhangt met een hogere kans op een positieve SARS-CoV-2-testuitslag dan het buiten sporten (OR 1,8; 95%-BI: 1,2–2,6; *p* = 0,003). Wanneer we specifiek kijken naar COVID RADAR-gebruikers die alleen maar binnen hadden gesport was er geen significant verschil met de hoofdanalyse. Er kon na correctie geen significant verband worden aangetoond tussen het aantal dagen binnen sporten en de testuitslag.

Deze bevinding komt overeen met onderzoek onder jonge atleten dat eerder in Amerika is uitgevoerd [1]. Daar werd ook een verband gevonden tussen binnen sporten en de besmettingskans met SARS-CoV‑2. Uit data van de COVID RADAR-app blijkt dat dit dus ook geldt voor de Nederlandse situatie en andere leeftijdscategorieën. Een verschil tussen het Amerikaanse onderzoek en het huidige onderzoek is de meting op individueel niveau en ook het temporele aspect binnen de COVID RADAR-app. Alleen in de COVID RADAR-app is het mogelijk om de relatie tussen sporten en testuitslag te onderzoeken in het meest waarschijnlijke tijdvak van mogelijke besmetting. Andere sterke punten van deze analyse zijn de correcties voor overig risicogedrag en de achtergrondprevalentie van SARS-CoV‑2.

De in deze analyses geselecteerde gebruikers van de COVID RADAR-app zijn gemiddeld ouder dan de Nederlandse bevolking. Mogelijke oorzaken hiervoor hebben we besproken in onze eerdere publicatie [2]. Logischerwijs vertonen ouderen minder risicogedrag, aangezien zij weten dat ze een hogere kans hebben dat een infectie met SARS-CoV‑2 ernstiger verloopt. Toch blijkt het percentage van de gebruikers dat wekelijks sportte goed overeen te komen met dat uit andere statistieken van Nederlandse sportdeelname. Ook het percentage dat binnen sportte was vergelijkbaar met Nederlandse statistieken [2]. Deze bevinding ondersteunt de externe validiteit van de gevonden verbanden.

Mogelijke beperkingen van deze analyse zijn de volgende. Het betreffen patiëntgerapporteerde uitkomsten, met alle daarmee gepaard gaande mogelijkheden van bias. De precieze testdatum is binnen de app niet gerapporteerd, maar uit onze eerdere publicatie passen het patroon van de symptomen en het gedrag rond de rapportage van een positieve test bij een correcte rapportage [2]. Gebruikers worden gevraagd een testuitslag van een PCR-test te rapporteren, maar het is mogelijk dat gerapporteerde testuitslagen ook van antigeen (zelf)tests afkomstig zijn. Door een periode te selecteren van meerdere dagen is het mogelijk dat een gebruiker in de periode voor een testuitslag zowel binnen als buiten sportte. Maar in de subanalyse met gebruikers die alleen maar binnen sportten ten opzichte van gebruikers die alleen maar buiten sportten was het effect vergelijkbaar. Door in de analyse alleen gebruikers te includeren met een bekende testuitslag worden mogelijk asymptomatische SARS-CoV-2-dragers geëxcludeerd, aangezien deze groep een lagere kans heeft om getest te worden. In onze eerdere publicatie is echter te zien dat 30% procent van de positief geteste COVID RADAR-gebruikers asymptomatisch is op de dag van de positieve SARS-CoV-2-test [2]. Dit stemt overeen met eerder onderzoek [9, 10].

Als laatste beperking moet vermeld worden dat het hier gaat over observationele data. Daarom kunnen alleen verbanden getest worden en kunnen er geen definitieve conclusies over causaliteit getrokken worden. We hebben hiervoor geprobeerd te corrigeren door een logische periode (de periode van mogelijke besmetting) voor een testuitslag te kiezen.

Ook de subanalyse en overige correcties die we hebben uitgevoerd vormen sterke kanten van deze analyse en wijzen in de richting van een oorzakelijk verband. Het was niet mogelijk om te corrigeren voor andere confounders (bijvoorbeeld comorbiditeit) in de relatie tussen een positieve test en het wel of niet buiten sporten. Daarom kunnen geen uitspraken gedaan worden over de impact die deze hogere kans op besmetting met SARS-CoV‑2 op de zorgvraag zou kunnen hebben. Ook waren er geen gegevens bekend over het soort sport dat werd beoefend. Mogelijk is er een verschil tussen teamsporters en individuele sporters wat betreft de kans op besmetting met SARS-CoV‑2.

## Conclusie

Uit de analyse van data die met de COVID RADAR-app werden verzameld, blijkt dat wanneer iemand sport, binnen sporten samenhangt met een hogere kans op een positieve testuitslag ten opzichte van het buiten sporten. Dit bevestigt de rationale achter het eerdere beleid ten aanzien van het langer gesloten houden van binnensportaccommodaties om het aantal besmettingen te reduceren.
