# Supplementary material for: Indoor sporting during the COVID-19 pandemic: analysis with data from the COVID RADAR app
Source: TSG. 2022 Jun 7;100(3):92–7. [Article in Dutch] doi: 10.1007/s12508-022-00351-0 (PMC9172613; doi:10.1007/s12508-022-00351-0)
Supplement: Supplementary file 2 [file 12508_2022_351_MOESM2_ESM.docx]

**Bijlage 2**

**Tabel B1** **Positieve en negatieve Coronatest per leeftijdsgroep**

|  | Negatief | % | Positief | % |
| --- | --- | --- | --- | --- |
| 0-18 | 54 | 72 | 21 | 28 |
| 19-49 | 177 | 86,8 | 27 | 13,2 |
| 50-69 | 718 | 87,4 | 104 | 12,7 |
| 70+ | 227 | 90,1 | 25 | 9,9 |
|  | 1176 | 87 | 177 | 13 |
