# Supplementary material for: Indoor sporting during the COVID-19 pandemic: analysis with data from the COVID RADAR app
Source: TSG. 2022 Jun 7;100(3):92–7. [Article in Dutch] doi: 10.1007/s12508-022-00351-0 (PMC9172613; doi:10.1007/s12508-022-00351-0)
Supplement: Supplementary file 3 [file 12508_2022_351_MOESM3_ESM.docx]

**Bijlage 3**

**Tabel B2** **Positieve en negatieve test ten opzichte van alleen binnen sporten of alleen maar buiten sporten**

|  | Alleen buiten sporten | % | Alleen binnen sporten | % |
| --- | --- | --- | --- | --- |
| Negatief | 705 | 91,7 | 396 | 80,3 |
| Positief | 64 | 8,3 | 97 | 19,7 |
| Totaal | 769 |  | 493 |  |
