# Supplementary material for: Indoor sporting during the COVID-19 pandemic: analysis with data from the COVID RADAR app
Source: TSG. 2022 Jun 7;100(3):92–7. [Article in Dutch] doi: 10.1007/s12508-022-00351-0 (PMC9172613; doi:10.1007/s12508-022-00351-0)
Supplement: Supplementary file 4 [file 12508_2022_351_MOESM4_ESM.docx]

**Bijlage 4**

**Tabel B3.** **Positieve en negatieve tests per aantal dagen binnensporten**

|  | Negatief | % | Positief | % |
| --- | --- | --- | --- | --- |
| 0 | 705 | 91,7 | 64 | 8,3 |
| 1 | 310 | 79,7 | 79 | 20,3 |
| 2 | 105 | 82 | 23 | 18 |
| 3 | 37 | 82,2 | 8 | 17,8 |
| 4 | 10 | 83,3 | 2 | 16,7 |
| 5 | 3 | 75 | 1 | 25 |
| 6 | 4 | 100 | 0 | 0 |
| 7 | 1 | 100 | 0 | 0 |
| 8 | 0 |  | 0 |  |
| 9 | 1 | 100 | 0 | 0 |
| Totaal | 1176 | 86,9 | 177 | 13,1 |
