# Supplementary material for: Indoor sporting during the COVID-19 pandemic: analysis with data from the COVID RADAR app
Source: TSG. 2022 Jun 7;100(3):92–7. [Article in Dutch] doi: 10.1007/s12508-022-00351-0 (PMC9172613; doi:10.1007/s12508-022-00351-0)
Supplement: Supplementary file 5 [file 12508_2022_351_MOESM5_ESM.docx]

Bijlage 5

Tabel B4. Hoofdanalyse; minimaal 1 maal binnensporten versus niet binnensporten

| **Univariaat** | **OR** | **P** | **95% CI low** | **95% CI high** |
| --- | --- | --- | --- | --- |
| Minimaal 1 maal binnensporten | 2,64 | <0,001 | 1,91 | 3,65 |
| **Multivariaat** |  |  |  |  |
| Minimaal 1 maal binnensporten | 1,78 | 0,003 | 1,21 | 2,61 |
| Leeftijd (19-49) | 0,31 | 0,002 | 0,15 | 0,65 |
| Leeftijd (50-69) | 0,24 | <0,001 | 0,13 | 0,46 |
| Leeftijd (70+) | 0,16 | <0,001 | 0,07 | 0,34 |
| Leefbarometer | 1,99 | 0,239 | 0,63 | 6,2 |
| Gemiddeld aantal personen <1,5m | 0,99 | 0,47 | 0,96 | 1,02 |
| Gender | 0,99 | 0,952 | 0,68 | 1,43 |
| Vaccinatiestatus | 1,48 | 0,07 | 0,97 | 2,26 |
| PeriodePrevalentie | 1,001 | <0,001 | 1,0010 | 1,0016 |

Tabel B5. Subanalyse 1: alleen binnensporten en alleen buitensporten

| **Univariaat** | **OR** | **P** | **95% CI low** | **95% CI high** |
| --- | --- | --- | --- | --- |
| Alleen binnensport | 2,70 | <0,001 | 1,93 | 3,7 |
| **Multivariaat** |  |  |  |  |
| Alleen binnensport | 1,84 | 0,003 | 1,23 | 2,75 |
| Leeftijd (19-49) | 0,34 | 0,006 | 0,16 | 0,73 |
| Leeftijd (50-69) | 0,26 | <0,001 | 0,13 | 0,50 |
| Leeftijd (70+) | 0,15 | <0,001 | 0,07 | 0,36 |
| Leefbarometer | 2,97 | 0,092 | 0,84 | 10,5 |
| Gemiddeld aantal personen <1,5m | 0,99 | 0,46 | 0,96 | 1,02 |
| Gender | 0,99 | 0,975 | 0,67 | 1,47 |
| Vaccinatiestatus | 1,4 | 0,11 | 0,92 | 2,23 |
| PeriodePrevalentie | 1,001 | <0,001 | 1,0010 | 1,0016 |

Tabel B6. Subanalyse 2: Verband met iedere dag extra binnensporten

| **Univariaat** | **OR** | **P** | **95% CI low** | **95% CI high** |
| --- | --- | --- | --- | --- |
| Aantal dagen binnensport | 1,29 | <0,001 | 1,14 | 1,46 |
| **Multivariaat** |  |  |  |  |
| Aantal dagen binnensport | 1,10 | 0,363 | 0,90 | 1,35 |
| Leeftijd (19-49) | 0,31 | 0,003 | 0,15 | 0,64 |
| Leeftijd (50-69) | 0,25 | <0,001 | 0,13 | 0,45 |
| Leeftijd (70+) | 0,15 | <0,001 | 0,07 | 0,34 |
| Leefbarometer | 1,82 | 0,297 | 0,59 | 5,6 |
| Gemiddeld aantal personen <1,5m | 0,99 | 0,566 | 0,96 | 1,02 |
| Gender | 0,97 | 0,896 | 0,67 | 1,4 |
| Vaccinatiestatus | 1,7 | 0,013 | 1,12 | 2,66 |
| PeriodePrevalentie | 1,001 | <0,001 | 1,0010 | 1,0016 |
